# Annual Nitrogen Balance from Dairy Barns, Comparison between Cubicle and Compost-Bedded Pack Housing Systems in the Northeast of Spain

**DOI:** 10.3390/ani11072136

**Published:** 2021-07-19

**Authors:** Esperanza Fuertes, Ahmad Reza Seradj, Jordi Maynegre Santaularia, Daniel Villalba Mata, Gabriel de la Fuente Oliver, Joaquim Balcells Teres

**Affiliations:** Department of Animal Science, Universitat de Lleida-Agrotecnio-CERCA Center, Alcalde Rovira Roure 191, 25198 Lleida, Spain; esperanza.fuertes@udl.cat (E.F.); jordi.maynegre@udl.cat (J.M.S.); daniel.villalba@udl.cat (D.V.M.); gabriel.delafuente@udl.cat (G.d.l.F.O.); joaquim.balcells@udl.cat (J.B.T.)

**Keywords:** nitrogen recovery, nitrogen loss, manure management, dairy housing

## Abstract

**Simple Summary:**

Environmental concerns related to livestock production have driven the importance of developing manure-management alternatives to mitigate N emissions. Hence, we carried out measurements of N loss in two different dairy housing systems (compost-bedded pack vs. cubicles), each system with a very distinct manure-management procedure. Since each system performs different manure-management practices, it may be important to compare their efficiency in terms of N recovery from manure in both systems. In contemplation of annual temperature variation, measurements of N recovery after manure management were carried out during warm and cold seasons of the year. Significantly higher volatilization rates were found in compost-bedded pack system with respect to cubicles system, therefore, N left in manure was lower in compost-bedded pack barns.

**Abstract:**

The aim of this study was to determine N recovery and irreversible losses (i.e., through NH_3_-N volatilization) from manure in two different housing systems throughout a year using an N mass balance approach. Dietary, milk, and manure N were monitored together with outside temperatures in six dairy barns during six months, comprising two different seasons. Three barns were designed as conventional free stalls (cubicle, CUB) and the other three barns as compost-bedded packs (CB). All the barns were located in the Ebro’s valley, in the northeast of Spain. Mass N balance was performed simultaneously in the six barns, during two three-month periods (Season I and II) and sampling at a 15-day interval. Results of ANOVA analysis showed that annual N retained in manure (kg/head per year) from cows housed in CUB barns was significantly higher than in manure from cows housed in CB (133.5 vs. 70.9, *p* < 0.001), while the opposite was observed for N losses (26.9 vs. 84.8, for CUB and CB barn, respectively; *p* < 0.005). The annual mean proportion of irreversible N loss from manure in relation to N intake was much lower in barns using conventional free-stall cubicles than the mean ratio registered in bedded pack systems barns.

## 1. Introduction

Manure application in croplands poses potential risks to the environment, not just for its concentration in toxic metals and potentially pathogenic bacteria [1,2], but mainly for the impact produced by the excess of N upon the water, soil, and atmosphere [3,4]. Compost-bedded pack barns are alternative to conventional housing cubicles for dairy cows. In these barns, the entire resting area consists of a deep bedded pack dairy stirred in order to favor the water evaporation and composting process [5]. Potential advantages of compost-bedded pack rely on animal welfare and manure management [6]. However, there is still little scientific evidence of its environmental impact in relation to those data generated under conventional cubicles facilities [7].

The European Directive 91/676/EC aimed to reduce soil and water pollution from nitrates used for agriculture, thus, it has been necessary to develop a reliable definition of standard values for N in manure from different livestock species. The European Commission proposed that N excretion would be quantified as the difference between N intake and N retention in the different animal’s products; thus, N in manure should be calculated assuming a constant percentage of N lost in the atmosphere during waste removal and storage following the methodology proposed by ERM/AB-DLO [8].

Currently, Environmental Resources Management (ERM) reference data are based on North Europe information, but it is well-known that N in manure may differ among European countries and locations [9,10,11]. Consequently, it is imperative to precisely describe N manure production under different climate scenarios and husbandry systems. Dairy cattle are relatively inefficient dietary N utilizers, as the efficiency of transfer of feed N into milk protein N has been determined to be about 0.23, being the remaining N excreted through urine and feces [12]. Once this N has been excreted and both droppings have been mixed, volatilization mainly through NH_3_, known for its negative impacts on ecosystems and health, can occur easily if conditions are favorable [13,14]. Therefore, the objective of this study is to quantify, including the lactating and non-lactating period, the N mass balance (kg N/year per head) in dairy cattle barns through an annual cycle comparing two housing systems, conventional solid floor cubicle (CUB; where cows are confined in concrete floor cubicles, manure being collected daily from the feed alley and stored in an open air concrete pool [15]) and compost-bedded pack (CB; where an open resting area free of partitions made from the cattle manure is provided, which is daily composted “in situ” by the tillage of a rotary harrow or cultivator) [16].

## 2. Materials and Methods

### 2.1. Study Setting and Animal Management

In the present study, both types of farms were located in the same area, by the Ebro’s valley in the northeast of Spain. The case studies were run simultaneously in six commercial dairy cattle barns using different housing facilities; three were equipped with a conventional free-stall system using cubicles (CUB), and the other three were equipped with a loose housing system with a compost-bedded pack (CB), provided with a feed alley that was mechanically cleaned 2–3 times per day. All farms raised Holstein Friesian cows from 1 to 4 parturitions and between housing system no differences between CUB and CB were detected in either age (4.1 and 4.2 years respectively; SE = 0.06) nor parturition interval (426 and 438 days respectively; SE = 4.9), although mean lactation number was slightly higher in cows managed in CUB barns (2.43 vs. 2.23; SE = 0.029). Cows were artificially inseminated (at ≈ 157 days after calving), and dried-off 70 days before the next calving; on average, days in milk (DIM) were 83.6% [17] while the rest of the year (16.4%) cows were under non-lactating phase. Details of the lactating-phase and temperatures registered during sampling are available in Balcells et al., 2020 [18].

Non-lactating cows [averaging 36 and 42 cows for CUB and CB systems, respectively (SE = 10.9)] were managed together with replacement heifers, and housed in an outdoor paved area with partially covered pens. Stocking rate ranged from 10 to 25 m^2^/cow and no bedding material was used. No weight recording facilities were available so a standard-constant weight for non-lactating/pregnant cows of 725 kg was assumed.

### 2.2. Experimental Diet

Dry cows and replacement heifers were feed “ad libitum” a total mixed ration (TMR) based on grass silage plus straw, occasionally supplemented with either soya or rapeseed meal. Dry matter and N intake during the non-lactating period was established based on ratio composition and energy requirements for maintenance (for 725 kg Live weight cow) plus pregnancy, considering an average post-conception period of 245 days and using 40 kg calf weight at calving. Energy requirements were estimated in accordance with the Agricultural and Food Research Council [19].

### 2.3. N Excretion and N Loss Calculation

Total N excretion was calculated as the difference between N intake and N retention in the fetus milk assuming that lactating cows fully recovered their body condition in the last part of the lactation phase. N lost in the atmosphere during manure storage was calculated by subtracting the amount of N remaining in the manure after storage from total N excretion. N loss measurements were performed during two three-month periods; Season I (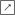
Tª: from February to May) and Season II (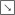
Tª: from August to November); applying in both periods same sampling protocol. Within each season, through every 15-days sampling intervals manure deposited into the pens was cleaned mechanically using a tractor provided with a shovel and deposited, sampled (25–50 spot samples, 0.5–1 kg FM each), and stored in a dung-heap. CUB and CB barns were sampled in alternate weeks. To assess the theoretical Manure-N losses, a manure pool simulator (MPS) was designed, consisting of a plastic barrel (85 cm diameter and 160 cm height) placed near the dung-heap of each farm under study to replicate the same environmental conditions. Every sampling day (i = 1 to 6) N stored in the MPS (N stored _MPSi_) was calculated as the sample weight (20–40 kg FM) multiplied by the N concentration. At the end of each season, total MPS manure was weighed, sampled, and analyzed to calculate N left in the MPS (N left_MPS_). Differences between N stored in the MPS, calculated as the summation $\overset{6}{\underset{i=1}{\sum }}\left(N\;\mathrm{stored}\;\mathrm{MPS}i\right),$ and N left_MPS_ were considered a valid index of the N losses from the manure stored in the dung heap. An MPS-loss coefficient (k) in grams of N lost/gram of N stored was calculated as follows:

(1) $$k=\frac{\sum_{i=1}^{6}\left(N\;\mathrm{storedMPS}i\right)-N\;\mathrm{leftMPS}}{\sum_{i=1}^{6}\left(N\;\mathrm{storedMPS}i\right)}$$

finally, N left in the manure (g/d per cow) = N excretion × (1 − k);

On the sampling day, the offered feed was sampled, dried for 48 h at 60 °C in a forced-air oven, ground (1-mm screen diameter), and analyzed for DM and N; N was determined by the Kjeldahl method [20]. Physicochemical composition of the milk (fat, protein, lactose, and total solid content) was determined by the Dairy Inter-Professional Association of Catalonia testing service (ALLIC) using MilkoScan™ equipment. Manure from MPS was weighed, thawed, and density was measured by the weight/volume ratio. Fresh samples were divided into two sub-samples to analyze total N and NH_3_-N; NH_3_-N was determined by direct distillation with Na_2_B_4_O_7_ [21].

### 2.4. Statistical Analysis

The barn was considered the experimental unit and data were analyzed with the MIXED procedure in SAS (v9.4) using the following model: Y_ijkl =_ µ + HS_i_ + F_j_ + S_k_ + (HS × S)_ijk_ + ε _ijkl_; Y_ijkl_ is a measurement from a herd of cows allocated in each barn, µ is the overall mean, HS_i_ is the manure-management system (CUB; CB), F_j_ is the fortnightly effect and is treated as a repeated measure (j = 1 to 6). S_k_ is the seasonal effect (Season I, increasing temperature; Season II, decreasing temperature) plus interactions and ɛ_ijkl_ is the residual error. Tukey multiple comparison test was applied, and significant differences and trends were declared at *p* ≤ 0.05 and 0.05 < *p* ≤ 0.10, respectively.

## 3. Results and Discussion

Authors are unaware of data comparing annual N mass balance (including the lactating and non-lactating period) between the analyzed dairy barns systems (i.e., kg N/year per head), but a comprehensive comparison referring only to lactating cows is shown in [18]. Briefly, data of N balance generated within conventional cubicles system fit well within meta-analysis published by [22] and fall within the interval of irreversible N losses (72 to 129 g N/cow per day) proposed by several authors [14,23,24,25] when specific data from the mass N balance in conventional solid floor cubicle system were considered (building facilities plus manure storage pools). Data related to irreversible N losses coming from CB system are scarce, our data (Table 1) fit well into the range of irreversible N losses coefficient (N losses/manure N produced) proposed by Rotz (2004) [26] 35%, and Atzori et al. (2009) [7] 38.8%.

In relation to the season, a positive correlation between temperature and NH_3_ volatilization has been described by several authors [27,28], although, in the present study, differences between average temperatures (16.5 vs. 12.3 °C, for season I and II, respectively) were probably small to discriminate potential changes in N losses.

In order to calculate annual N production through a complete year cycle (kg N/year head) (Table 2), data from lactating [18] and non-lactating cows (Table 1) were combined, considering an averaged value of days in milk (DIM) of 83.6% of the whole year. Experimental data collected in six months (two three-month season) were extrapolated to a one-year cycle. At this point is necessary to remark that CUB barns combine cycles of manure storage with land application no longer than 3 months, so longer manure storage periods are not usual in the conventional system managing. Authors are aware that the extrapolation process might add some degree of uncertainty to the calculations, but is also true that year-to-year variation (by land and/or weather conditions) does exist, then a whole year projection also lay open to unpredictability.

Although the non-lactating animals housed in CUB systems numerically showed higher N ingestion (g/d; 266.4 vs. 234.3, *p* = 0.14) and N left in the manure (218.1 vs. 186.7, *p* = 0.11) than those housed in CB barns, but the differences did not reach statistical significance. The season did not influence the N intake, but the irreversible N losses to the atmosphere tended to be higher during the warm season (Table 1). This could be explained by the fact that higher temperatures would increase NH_3_ emission rates, as urease activity increases exponentially above 10 °C [29], given that the main way of N volatilization from manure is through this gas [30,31,32]. 

When it comes to the annual N balance, neither differences in N intake nor N excreted through the milk were observed between housing systems, although annual N left in manure (kg/head per year) from cows housed in CUB barns was significantly higher compared to those housed in CB barns (133.5 vs. 70.9, *p* < 0.001), while the opposite was observed for the irreversible N losses to the atmosphere, CB system being the one with higher N volatilization (26.9 vs. 84.8, for CUB and CB barn, respectively; *p* < 0.005). This could be explained due to the aeration process generated daily in CB barns when composting is performed in the bed, resulting in aerobic conditions that favor and enhance N volatilizations, mainly through NH_3_ [33,34]. As a result of the high N volatilization produced in CB barns, its net N recovery in manure was much lower than a conventional free-stall CUB barn. The annual mean proportion of irreversible N loss from manure in relation to N intake was much lower in barns using conventional free-stall cubicles (12.3%) than the mean ratio registered in bedded-pack systems barns (40.2%).

Nitrogen intake from the diet was close to the average (227.45) reported [35], where cows in Season I ate more N (223.6 vs. 204.5; *p* < 0.004) and apparently excreted more N through milk (62.5 vs. 53.8 kg/head per year; *p* < 0.2) than in Season II. Fresh manure production (Tn/head/year) was higher in animals housed in CUB systems (33.5 vs. 10.3 for CUB and CB, respectively) although, in terms of dry matter, differences were much smaller between housing systems (3.48 vs. 3.03 for CUB and CB, respectively; *p* = 0.001) and higher between seasons (2.85 vs. 3.53; for Season I and II, respectively; *p* = 0.05).

Mean of manure N produced or in other words, gross N recovery (N diet-N in milk; kg N/year) of both systems was 156 kg, with average retention in milk (N utilization efficiency) of 27% and was within the range (12.6–36.2%) reported previously [35]. Mean net N recovery (N diet—N in milk-N losses from buildings and manure storage; kg N/year) reached 102 kg, with a Milk-N value close to 30%.

The mean of manure N storage efficiency (N left into manure/manure N produced), in both systems, was 65% which was higher than 26% reported previously [35] and probably is because of less N volatilization at the farm level, due to frequent manure removal as suggested in other studies [9,26].

Subsequently, the mean of whole-farm N efficiency in both systems (N in total output divided by N in total input) is higher.

On the other hand, irreversible N losses coefficient (N losses/manure N produced), in both systems was 35% and was in the upper range (24 to 34% of N losses) of the results provided from Belgium barns [36] and lower than 39% of N volatilization [37] during storage.

Gross N recovery values were, in general, lower than those reported by the National Greenhouse Gas Inventories [38] for which most state members applied country-specific approaches for their N excretion estimates. In the case of dairy cattle, this estimation ranged from less than 80 kg N/cow per year to over 140 kg N/cow per year. Under this scenario, our results are in the upper range, although the values proposed in the CAPRI (Common Agricultural Policy Regional Impact) model [39,40] were much higher, e.g., 194 or 180 kg N kg N/cow per year for Denmark and Sweden, respectively. In relation to the specific framework from the EU Nitrate Directive (2011) based on Ketelaars and Van Der Meer [41] criteria, it is indicated that in Spain, the theoretical net N recovery for manure is 89 kg N/cow per year. However, other countries such as the Netherlands (from 99 to 131 kg N/cow per year), Sweden (117–139 kg N/cow per year), or Germany (100 to 149 kg N/cow per year) have proposed much greater and consistent values.

The relevant differences in the estimation of N recovery (gross/net N recovery) in dairy cattle among EU members are linked to two primary issues: first, variations in methodology, by collecting and combining the data in the different policy reports [42]; and second, variations linked to the cow breed, dietary N content, milk production [41], and also manure management [42,43].

## 4. Conclusions

In the dairy farms, the principal issue is to deal with is the efficiency of the dairy cows to transform feed N into milk N, improving net N recovery and diminishing N wastes. Our results confirm the impact of the manure-management and/or housing system on mass N balance in dairy farms and should be considered in future models to precisely predict N waste from animal manure. Based on our findings, the net N recovery and the N utilization efficiency in a conventional free stall (CUB: 133.5 kg/year and 27.5%, for N recovery and N utilization efficiency, respectively) were higher than that of a compost-bedded pack (CB: 70.9 kg/year and 26.8%, for N recovery and N utilization efficiency, respectively).

## Figures and Tables

**Table 1:** N balance (g/head/day) and N emission in dry-pregnant cows in two housing systems and two seasons.

Variables (g/Head/Day)	Housing System ^1^		Season ^2^		RSD ^3^	*p*-Value ^4^		
	CUB	CB	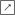 Tª	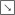 Tª		HS	S	HS × S
N intake	266.4	234.3	251.8	251.4	30.16	0.14	0.82	0.83
Theoretical N Retention into fetus	13.7	13.7	13.7	13.7	-	-	-	-
Theoretical N left in manure	218.1	186.7	201.3	203.5	26.12	0.11	0.85	0.9
Theoretical irreversible N losses	34.36	33.9	36.4	34	1.64	0.57	0.07	0.88

^1^ CUB = cubicle; CB = compost-bedded pack.^2^ 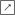
Tª = increasing temperature: from February to May; 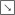
Tª = decreasing temperature: from August to November. ^3^ RSD = residual standard deviation. ^4^ HS = housing system; S = season; HS × S = interaction of housing system and season.

**Table 2:** Annual N balance (kg/head/year) and total fresh and dry manure production (considering 83.6% days in milk) in two housing systems and two seasons.

N Balance (kg/Head/Year)	Housing System ^1^		Season ^2^		RSD ^3^	*p*-Value ^4^		
	CUB	CB	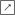 Tª	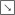 Tª		HS	S	HS × S
N intake	218.5	209.6	223.6	204.5	8.20	0.12	0.004	0.06
N milk	60.1	56.2	62.48	53.8	5.56	0.23	0.02	0.24
N fetus	0.82	0.82	0.82	0.82	-	-	-	-
N left into manure	133.5	70.9	98.4	106.0	17.27	0.0002	0.46	0.79
N Losses	26.9	84.8	64.9	46.8	20.52	0.0012	0.16	0.63
Fresh Manure (Tn/year per cow)	33.5	10.3	21.1	22.8	6.52	0.001	0.05	0.08
Dry Manure (Tn/year per cow)	3.48	3.02	2.85	3.53	0.52	0.07	0.04	0.30

^1^ CUB = cubicle; CB = compost-bedded pack. ^2^ 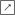
Tª = increasing temperature: from February to May; 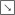
Tª = decreasing temperature: from August to November. ^3^ RSD = residual standard deviation. ^4^ HS = housing system; S = season; HS × S = interaction of housing system and season.

## Data Availability

Not applicable.

## References

[1] Hejna M., Moscatelli A., Onelli E., Baldi A., Pilu S., Rossi L. (2019). Evaluation of concentration of heavy metals in animal rearing system. Ital. J. Anim. Sci..

[2] Vukobratović M., Lončarić Z., Kerovac D. (2014). Heavy metals in animal manure and effects of composting on it. Acta Hortic..

[3] Rayne N., Aula L. (2020). Livestock Manure and the Impacts on Soil Health: A Review. Soil Syst..

[4] Sanchis E., Calvet S., del Prado A., Estellés F. (2019). A meta-analysis of environmental factor effects on ammonia emissions from dairy cattle houses. Biosyst. Eng..

[5] Klaas I.C., Bjerg B., Friedmann S., Bar D. (2010). Cultivated barns for dairy cows—An option to promote cattle welfare and environmental protection in Denmark?. Dan. Vettidsskr..

[6] Lobeck K., Endres M., Shane E., Godden S., Fetrow J. (2011). Animal welfare in cross-ventilated, compost-bedded pack, and naturally ventilated dairy barns in the upper Midwest. J. Dairy Sci..

[7] Atzori A.S., Boe R., Carta P., Fenu A., Spanu G., Francesconi A.H.D., Cannas A. (2009). Estimation of nitrogen volatilization in the bedded-pack of dairy cow barns. Ital. J. Anim. Sci..

[8] ERM/AB-DLO (1999). Establishment of Criteria for the Assessment of the Nitrogen Content of Animal Manures, European Commission, Final Report.

[9] Hou Y., Velthof G., Lesschen J.P., Staritsky I.G., Oenema O. (2016). Nutrient Recovery and Emissions of Ammonia, Nitrous Oxide, and Methane from Animal Manure in Europe: Effects of Manure Treatment Technologies. Environ. Sci. Technol..

[10] Sommer S.G., Webb J., Hutchings N. (2019). New Emission Factors for Calculation of Ammonia Volatilization from European Livestock Manure Management Systems. Front. Sustain. Food Syst..

[11] Webb J., Sørensen P., Velthof G., Amon B., Pinto M., Rodhe L., Salomon E., Hutchings N., Burczyk P., Reid J. (2013). An Assessment of the Variation of Manure Nitrogen Efficiency throughout Europe and an Appraisal of Means to Increase Manure-N Efficiency. Advances in Agronomy.

[12] Correa-Luna M., Donaghy D., Kemp P., Schutz M., López-Villalobos N. (2020). Efficiency of crude protein utilisation in grazing dairy cows: A case study comparing two production systems differing in intensification level in New Zealand. Animals.

[13] Bittman S., Mikkelsen R. (2009). Ammonia Emissions from Agricultural Operations: Livestock. Better Crops.

[14] Hristov A. (2011). Technical note: Contribution of ammonia emitted from livestock to atmospheric fine particulate matter (PM_2.5_) in the United States. J. Dairy Sci..

[15] Galama P., Ouweltjes W., Endres M., Sprecher J., Leso L., Kuipers A., Klopčič M. (2020). Symposium review: Future of housing for dairy cattle. J. Dairy Sci..

[16] Black R., Taraba J., Day G., Damasceno F., Bewley J. (2013). Compost bedded pack dairy barn management, performance, and producer satisfaction. J. Dairy Sci..

[17] FEFRIC (2018). Control Lleter de la Raça Frisona a Catalunya 2018.

[18] Balcells J., Fuertes E., Seradj A.R., Maynegre J., Villalba D., De La Fuente G. (2020). Study of nitrogen fluxes across conventional solid floor cubicle and compost-bedded pack housing systems in dairy cattle barns located in the Mediterranean area: Effects of seasonal variation. J. Dairy Sci..

[19] Agricultural and Food Research Council (1993). Energy and protein requirements of ruminants. An Advisory Manual Prepared by the Agricultural Food and Research Council Technical Committee on Responses to Nutrients.

[20] AOAC International (2010). Official Methods of Analysis of Association of Official Analytical Chemists.

[21] Goldman F.H., Jacobs M.B. (1953). Chemical Methods in Industrial Hygiene.

[22] Huhtanen P., Hristov A.N. (2009). A meta-analysis of the effects of dietary protein concentration and degradability on milk protein yield and milk N efficiency in dairy cows. J. Dairy Sci..

[23] Aguerre M.J., Wattiaux M.A., Hunt T., Larget B.R. (2010). Effect of dietary crude protein on ammonia-N emission measured by herd nitrogen mass balance in a freestall dairy barn managed under farm-like conditions. Animal.

[24] Cole N.A., Todd R.W. Nitrogen and phosphorus balance of beef cattle feedyards. Proceedings of the Texas Animal Manure Management Issues Conference.

[25] Moreira V.R., Satter L.D. (2006). Effect of Scraping Frequency in a Freestall Barn on Volatile Nitrogen Loss from Dairy Manure. J. Dairy Sci..

[26] Rotz C.A. (2004). Management to reduce nitrogen losses in animal production. J. Anim. Sci..

[27] Smits M., Valk H., Elzing A., Keen A. (1995). Effect of protein nutrition on ammonia emission from a cubicle house for dairy cattle. Livest. Prod. Sci..

[28] Kroodsma W., Veld J.W.H.H., Scholtens R. (1993). Ammonia emission and its reduction from cubicle houses by flushing. Livest. Prod. Sci..

[29] Powell J.M., Misselbrook T., Casler M.D. (2008). Season and Bedding Impacts on Ammonia Emissions from Tie-stall Dairy Barns. J. Environ. Qual..

[30] Sun L., Wu Z., Ma Y., Liu Y., Xiong Z. (2018). Ammonia volatilization and atmospheric N deposition following straw and urea application from a rice-wheat rotation in southeastern China. Atmos. Environ..

[31] Groenestein C., Hutchings N., Haenel H., Amon B., Menzi H., Mikkelsen M., Misselbrook T., van Bruggen C., Kupper T., Webb J. (2019). Comparison of ammonia emissions related to nitrogen use efficiency of livestock production in Europe. J. Clean. Prod..

[32] Grant R.H., Boehm M.T. (2020). Ammonia emissions from differing manure storage facilities at two midwestern free-stall dairies. Atmosphere.

[33] Blanes-Vidal V., Hansen M., Pedersen S., Rom H. (2008). Emissions of ammonia, methane and nitrous oxide from pig houses and slurry: Effects of rooting material, animal activity and ventilation flow. Agric. Ecosyst. Environ..

[34] Samer M., Ammon C., Loebsin C., Fiedler M., Berg W., Sanftleben P., Brunsch R. (2012). Moisture balance and tracer gas technique for ventilation rates measurement and greenhouse gases and ammonia emissions quantification in naturally ventilated buildings. Build. Environ..

[35] Spears R., Kohn R., Young A. (2003). Whole-Farm Nitrogen Balance on Western Dairy Farms. J. Dairy Sci..

[36] Pollet I., Christiaens J., Van Langenhove H. (1998). Determination of the Ammonia Emission from Cubicle Houses for Dairy Cows Based on a Mass Balance. J. Agric. Eng. Res..

[37] Hollmann M., Knowlton K., Hanigan M. (2008). Evaluation of Solids, Nitrogen, and Phosphorus Excretion Models for Lactating Dairy Cows. J. Dairy Sci..

[38] IPCC (2014). Climate Change 2014, Synthesis Report (Vol. 9781107025).

[39] Leip A., Britz W., Weiss F., de Vries W. (2011). Farm, land, and soil nitrogen budgets for agriculture in Europe calculated with CAPRI. Environ. Pollut..

[40] Leip A., Commission E., Wassenaar T., Commission E., Fellmann T., Commission E. (2010). Evaluation of the Livestock Sector’s Contribution to the EU Greenhouse Gas Emissions—Final Report.

[41] Ketelaars J.J.M.H., Van der Meer H.G. (1999). Establishment of Criteria for the Assessment of the Nitrogen Content of Animal Manures. Phase 2. Report to the Directorate General XI of the European Commission.

[42] Velthof G.L., Hou Y., Oenema O. (2015). Nitrogen excretion factors of livestock in the European Union: A review. J. Sci. Food Agric..

[43] Bougouin A., Leytem A., Dijkstra J., Dungan R.S., Kebreab E. (2016). Nutritional and Environmental Effects on Ammonia Emissions from Dairy Cattle Housing: A Meta-Analysis. J. Environ. Qual..

